# Spallation Characteristics of Single Crystal Aluminum with Copper Nanoparticles Based on Atomistic Simulations

**DOI:** 10.3390/nano11102603

**Published:** 2021-10-03

**Authors:** Dong-Dong Jiang, Peng-Yu Chen, Pei Wang, An-Min He

**Affiliations:** 1 Institute of Applied Physics and Computational Mathematics, Beijing 100094, China; jiangdd@mail.ustc.edu.cn (D.-D.J.); cpyaaa@163.com (P.-Y.C.); wangpei@iapcm.ac.cn (P.W.); 2 Graduate School of China Academy of Engineering Physics, Beijing 100088, China

**Keywords:** nanocomposite, aluminum, inclusion, spall, shock response, microstructure, molecular dynamics

## Abstract

In this study, the effects of Cu nanoparticle inclusion on the dynamic responses of single crystal Al during shockwave loading and subsequent spallation processes have been explored by molecular dynamics simulations. At specific impact velocities, the ideal single crystal Al will not produce dislocation and stacking fault structure during shock compression, while Cu inclusion in an Al–Cu nanocomposite will lead to the formation of a regular stacking fault structure. The significant difference of a shock-induced microstructure makes the spall strength of the Al–Cu nanocomposite lower than that of ideal single crystal Al at these specific impact velocities. The analysis of the damage evolution process shows that when piston velocity *u_p_* ≤ 2.0 km/s, due to the dense defects and high potential energy at the interface between inclusions and matrix, voids will nucleate preferentially at the inclusion interface, and then grow along the interface at a rate of five times faster than other voids in the Al matrix. When *u_p_* ≥ 2.5 km/s, the Al matrix will shock melt or unloading melt, and micro-spallation occurs; Cu inclusions have no effect on spallation strength, but when Cu inclusions and the Al matrix are not fully diffused, the voids tend to grow and coalescence along the inclusion interface to form a large void.

## 1. Introduction

Spallation is a typical dynamic tensile failure process, which is caused by the tensile stress produced by the superposition of unloading wave and reflected sparse wave from free surface under shock loading [1]. It is of practical importance in virtually all applications involving rapid loading by explosives, impact, or energy deposition [2]. The predictive modeling of the experimentally observed behavior of metallic materials under shock loading conditions (wave structures, spall strengths) is a critical challenge toward the design of next-generation structural materials [3]. Earlier studies revealed that spallation is a complex multi-scale process [1,4], affected by many factors such as shock pressure [5], loading waveform [6], strain rate [7,8], temperature [9,10], and heterogeneity in the microstructure [11,12,13]. Compared to the loading conditions that can be directly controlled, it is more difficult to study the effect of microstructure on spallation due to the lack of high resolution and ultra-fast in-situ diagnostic technology. At the same time, due to the wide use of multiphase alloys and nanocomposites in engineering, it is very important to study the influence of inclusions on the spalling process.

In recent years, there were many experimental studies on the spallation of metals containing inclusions [14,15,16,17,18,19,20,21,22,23]. Work by Cheng et al. [14] on the effects of boron particles on the spallation of Al shows that the addition of boron particles in Al reduces spall strength by more than 30%, and the particle–matrix interfaces serve as the main void nucleation sites that dictate spall strength. Work by Minich et al. on single crystal Cu with SiO_2_ inclusions shows that the presence of SiO_2_ precipitates lowers the stress required to nucleate voids in this material as compared to that for pure Cu. Fensin et al. [17] found that the spall strength of Cu increases with the addition of Ag and Nb by 6% and 26%, respectively. The voids in the Cu–Nb alloy nucleate on the Cu/Nb interface, but the voids nucleation in the Cu–Ag alloy is independent of the interface, which is related to the hardness of the matrix and inclusions. In the study of Kanamori et al. [24], both the experimental and simulation results show that the strength of the Fe–Cu interface in Fe–Cu composite is lower than that of the matrix. In addition, the SEM images of the fracture surfaces in Al alloy [20,21] and steel [22] also have shown the influence of the interface between two phases in materials on spallation damage.

Even though there are a large number of experimental results, the mechanism of inclusions affecting spall strength and damage evolution is still not very clear. On the one hand, measurements on spall experiments are generally limited to monitoring the free surface velocity to infer the spallation strength [25] and recovering the spalling samples for microstructure characterization [20]. Although some direct measurements such as proton radiography [26], X-ray tomography [27], and situ femtosecond XRD [28] are also available, the microstructure and stress inside the material are still difficult to obtain. On the other hand, the influence of initial defects on the spallation process is not only the tensile fracture process but also the impact compression process of materials [29,30]. For the need of theoretical research, molecular dynamics (MD) simulation is widely used in the study of metal spallation [31,32,33,34]. Based on MD simulation, the in-situ observation from compression process to tensile process can be carried out, and we can directly obtain the local physical quantities such as stress, density, and temperature.

This paper focuses on the effect of Cu nanoparticles embedded in Al. In recent years, a number of experiments [35,36,37,38,39] and molecular dynamics [40,41] studies have been conducted on the mechanical and thermodynamic properties of Al–Cu alloys. Pogorelko et al. carried out a molecular dynamics study on the dynamic tensile process of single crystal Al containing Cu inclusion [42], and they found that the inclusion would reduce the dynamic tensile strength of the matrix due to the stress concentration effect. However, the response of Al–Cu nanocomposites under shock loading has not been studied in detail. In this work, a series of MD simulations were performed on single-crystal Al containing Cu nano-inclusions, and the effects of Cu inclusions on the microstructure during shock compression and spall damage were analyzed in detail under various shock intensities.

## 2. Methods and Simulation Details

**Figure 1:**
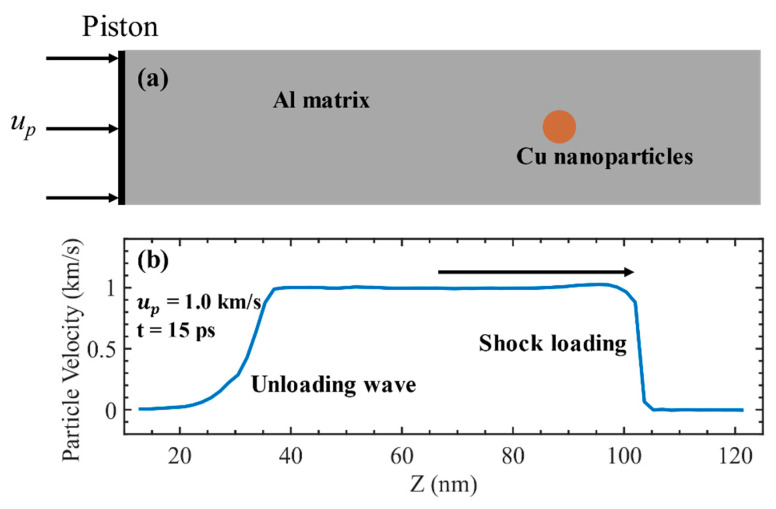
(**a**) Initial configuration of the simulation system. The matrix is single crystal Al and the spherical inclusion is single crystal Cu. (**b**) The loading profile at *u_p_* = 1.0 km/s and t = 15 ps.

The LAMMPS package [43] is used for MD simulation. We adopted the tabulated embedded-atom-method (EAM) potential by Zhakhovskii et al. [44] to describe the atomic interactions in Al. This potential was established to simulate the behavior of Al crystals under the strong shock conditions, and it has been successfully used in several studies related to the MD simulations of shockwave loading [11,45,46,47]. The interatomic interactions for Cu–Cu were described by the EAM potential developed by Mishin et al. [48]. An interatomic potential [49], which is an angular dependent potential (ADP), is a generalization of the EAM potential that has been used to describe the interatomic interactions for Al–Cu. The initial configuration of the simulation system is shown in Figure 1a. The Al single crystal of 80(x) × 80(y) × 300(z) FCC unit cells is constructed. The x, y, and z axes are, respectively, along the [100], [010], and [001] crystallographic directions. The lattice constant *a* of Al is 4.057 Å, and there are ~8 × 10^7^ atoms in the box. In the case of Al–Cu nanocomposite, a sphere of radius 3.6 nm is cut out in the single-crystal Al matrix, and a spherical inclusion of the same radius made from single-crystal copper is put inside the pore. Cu inclusion has the same lattice directions as those surrounding Al. Adjust the position of the inclusion at different piston velocities so that the inclusions are always located at the center of the spall plane. Before shock loading, the system is relaxed for 50 ps in NPT ensemble to reach an equilibrium state at 300 K and zero pressure. The shock compress and spallation processes are simulated by NEMD simulations with a time step of 1 fs. First, shock wave in the sample was generated by moving a piston at the left side of the target, as shown in Figure 1a. The piston velocity is also the particle velocity in the post-shocked region and is denoted as *u_p_*. The piston velocity kept constant for 12 ps. Then, the piston was removed to simulate the unloading process. The total simulation time was set to approximately 52 ps to observe the complete spall process. A typical resulting loading profile is shown in Figure 1b. Along the shock loading direction (Z-axis), free boundary condition is adopted and periodic boundary conditions are applied along the X- and Y-axis. Binning analysis with a bin width of 16 Å along the z-direction was applied to obtain local physical quantities such as stress and temperature. The atomic stress was calculated from the virial and thermal velocity. To quantify the microstructure evolution during shock compression and spallation, adaptive common neighbor analysis (a-CNA) [50] and Centro-symmetry parameter (CSP) [51] were used with the OVITO program [52]. In order to calculate the stress distribution near the inclusion, a layer of atoms with a thickness of 16 Å were selected in the central section of the sample *y* = *d*/2 (where *d* is the size in Y direction of MD model), then, a 2D-binning analysis with a bin size of 8 × 8 Å was used. The 3D-binning analysis method was used to analyze the void development. A three-dimensional grid of cubic cells was superimposed over the atomic configuration. Depending on the presence or absence of atoms in these cells, they are labeled as 0 or 1, resulting in a three-dimensional matrix. Clusters of two or more adjacent empty cells were identified as voids by solving the 3D-matrix for connected domains. The cell size was 5 Å. Periodic boundary conditions along X and Y directions were considered during the above analysis. Other authors have also adopted similar techniques [34,53,54].

## 3. Results and Analysis

### 3.1. Free Surface Velocity and Spall Strength

**Figure 2:**
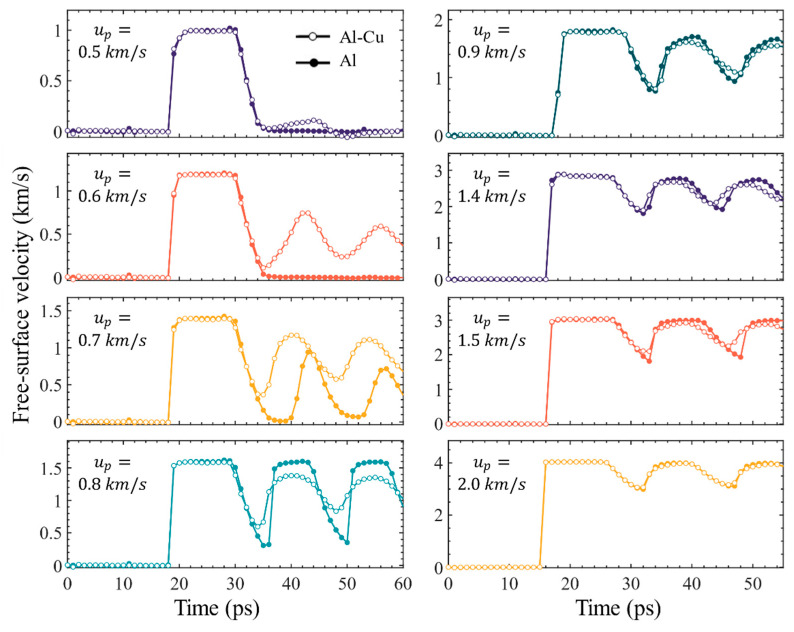
Time evolutions of free surface velocity for the crystal with Cu inclusion and the perfect crystal at various piston velocities. The solid circles represent the perfect crystal, and the open circles represent the crystal with inclusion.

Experimentally, a lot of information about spall process can be obtained from free surface velocity history. The free surface velocity histories for several cases are presented in Figure 2. Compared to the single crystal case, inclusions lower the critical piston velocity required for spall. When *u_p_* = 0.6 km/s, a clear spall signal appears in the inclusions case, while the single crystal case does not show any damage signal. When the piston velocity is 0.7 km/s, there is a clear signal of spall fracture for both the inclusions and single crystals cases, but the signal of spall fracture for single crystal is significantly later than that of the inclusions case. It can be seen that the free surface velocity is maintained near zero for a few ps before the pullback signal is generated, which means that the spall damage region is relaxed under the maximum tensile stress for a period of time before the damage fracture starts, i.e., 0.7 km/s is exactly the critical piston velocity for the single crystal case. It is clear that, when *u_p_* = 0.7 km/s and 0.8 km/s, the pullback velocities of ideal single crystal Al are greater than that of the sample with Cu inclusion. However, when 0.9 km/s ≤ *u_p_* ≤ 1.4 km/s, the pullback velocity of ideal crystal Al are roughly the same as that of the crystal with Cu inclusion, and when *u_p_* = 1.5 km/s, they are different again. When *u_p_* ≥ 2.0 km/s, Cu inclusions have almost no effect on the free-surface velocity history. In fact, the difference of pullback velocity reflects the influence of inclusions on spall strength.

**Figure 3:**
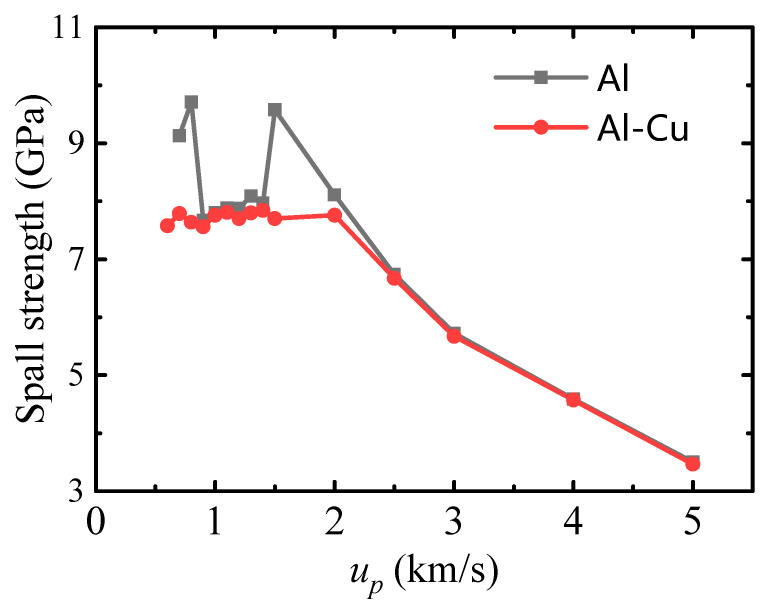
Spall strength at various piston velocities for the crystal with Cu inclusion and the perfect crystal.

Using the free surface velocity history and acoustic approximation, we can calculate the spall strength from the following expressions:

(1) $$\sigma_{sp}^{*}=0.5\rho_{0}c\Delta u_{fs}$$

where *ρ*_0_ is the initial density, and *c* and $\Delta u_{fs}$ are sound speed and the pullback velocity, respectively. This is a common method used in experiments to calculate the spall strength [20,55], while in molecular dynamics calculations, we can calculate the internal stress directly from the virial stress equation [56] and obtain the spall strengths $\sigma_{sp}$ from the maximum tensile stress. The spall strength calculated by these two methods at different piston velocities is given in Table 1. It can be seen that the results obtained by the two methods are basically consistent. The spall strength obtained from the internal stress calculation is adopted in the following discussion. As shown in Figure 3, for ideal single-crystal Al, the spall strength appears to vary non-monotonically and discontinuously at lower piston velocities due to the different shock compression structures at different piston velocities, as described in detail in a separate paper [57]. However, due to the presence of inclusions, the spall strength of Al–Cu nanocomposite keeps nearly constant at lower piston velocities. In other words, the inclusions significantly reduced the spall strength at *u_p_* < 0.9 km/s and 2.0 km/s > *u_p_* > 1.4 km/s, but they have little effect on the spall strength at piston velocities of 0.9–1.4 km/s. For higher piston velocities (*u_p_* ≥ 2.5 km/s), shock melting or unloading melting occurs, and the inclusions have almost no effect on the spall strength at all due to the temperature softening effect, the spall strength decreases with the increase of piston velocity.

Generally speaking, in addition to temperature and microstructure, tensile strain rate is an important factor affecting spall strength. Similar to spall strength, strain rate can also be calculated by free surface velocity history and acoustic approximation. The strain rate before fracture is given by:

(2) $$\dot{\varepsilon }=\frac{\Delta u_{fs}}{\Delta t}\frac{1}{2c}$$

where *c* is the sound speed, and $\Delta u_{fs}$ and Δt are the pullback velocity and the time associated with that pullback velocity, respectively. The strain rate at different piston velocities is given in Table 1. We found that, whether for single crystal Al or Al–Cu nanocomposites, the strain rate did not change significantly with the change of piston velocity. Therefore, we believe that, in this study, the spall strength was mainly affected by the microstructure evolution and temperature softening effect.

### 3.2. Shock Compression Process at Low Piston Velocities

**Figure 4:**
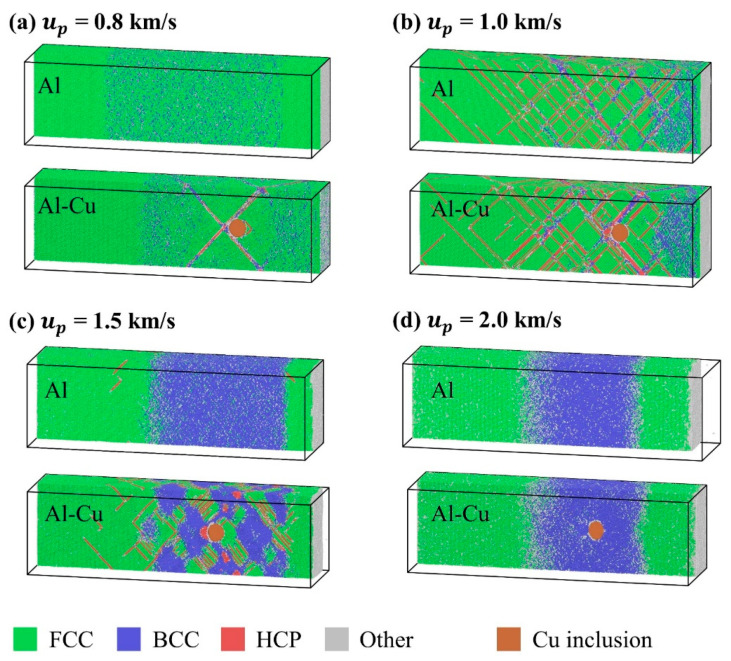
Atomic configurations during shock compression at 18 ps for different piston velocities as indicated in each figure. Al atom are color coded by adaptive common neighbor analysis (a-CNA). The cut surfaces of Cu inclusions are colored by the color of metallic copper. (**a**) *u_p_* = 0.8 km/s; (**b**) *u_p_* = 1.0 km/s; (**c**) *u_p_* = 1.5 km/s; (**d**) *u_p_* = 2.0 km/s.

Considering that the spall strength of single-crystal Al at lower piston velocities is directly controlled by the microstructural evolution during shock compression, the effect of inclusions on the spall strength should also be closely related to the microstructural evolution. Comparisons of microstructure evolution during shock compression of single-crystal Al and the sample with inclusions at different piston velocities are given in Figure 4. For the single crystal case, the characteristic of shock-induced microstructure can be divided into three parts, i.e., elastic deformation (*u_p_* ≤ 0.8 km/s), shock-induced dislocation and stacking fault (0.9 km/s ≤ *u_p_* ≤ 1.4 km/s), and shock-induced phase transition (1.5 km/s < *u_p_* < 3.0 km/s), which is the main reason for the variation of spall strength in the low piston velocity range in Figure 3, as analyzed in ref. [56]. However, due to the addition of inclusions, the characteristic of shock-induced microstructure changes considerably. For piston velocities *u_p_* ≤ 0.8 km/s, a regular stacking fault structure is formed on the four easiest glide surfaces tangent to the inclusion during shock compression. For 0.9 km/s ≤ *u_p_* ≤ 1.4 km/s, the shock-induced microstructure of single-crystal with inclusion is still characterized by dislocation and stacking fault, as in ideal single-crystal Al. When *u_p_* = 1.5 km/s, a homogeneous phase transition occurs in single-crystal aluminum, however, a complex structure of stacking faults and transformed phases are generated within the single-crystal Al containing inclusion. Combined with the spall strength in Figure 3, it can be seen that the inclusions have a significant effect on the spall strength only if there is no plastic deformation in the single crystal Al, but for the case where there is plastic deformation in both the single crystal Al and the single crystal Al with Cu inclusions during shock compression, the inclusions have no significant effect on the spall strength. Therefore, it is believed that the main way in which inclusions affect the spall strength is by affecting the microstructure during shock compression. In the following, we further analyze the evolution of the dislocation and stacking fault structure due to inclusions for two cases of piston velocity of 0.8 km/s and 1.5 km/s.

**Figure 5:**
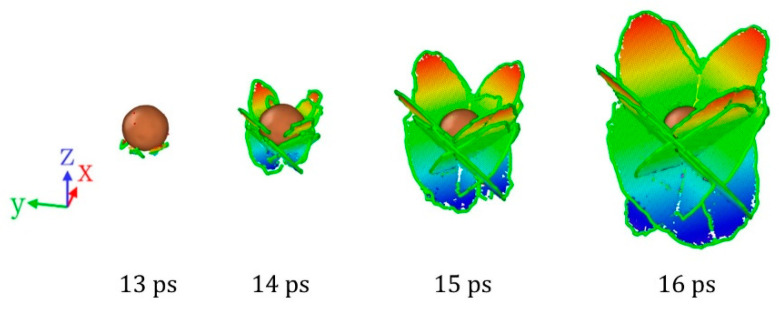
Dislocation and stacking fault structure near Cu inclusions at different times when *u_p_* = 0.8 km/s. The atoms are rendered by Z positions to enhance the stereoscopic effects.

In the case of ideal single crystal Al, the shear stress induced by the shock wave is not sufficient enough to generate dislocation in a short period of time, and therefore the sample remains as ideal single crystal when *u_p_* ≤ 0.8 km/s. However, for single crystal Al containing inclusions, when the inclusions and shock wave interact, four shear loops nucleate at the interface between inclusion and matrix, as shown in Figure 5. The Burgers vector of the leading partial dislocations of the four shear loops are 16[112],16[1¯12],16[11¯2],16[1¯1¯2], and the corresponding slip planes are $\left(111\right),\left(\bar{1}11\right),\left(1\bar{1}1\right),\left(\bar{1}\bar{1}1\right)$. As the shear loops expand further, the leading Shockley partial dislocations on two different {111} slip planes meet and interact, forming the dislocation and stacking fault structure with four-fold symmetry with respect to the loading direction, as illustrated in Figure 5. Almost identical stacking fault structures were also observed in the shock responses of single crystal Al with nano-porous [58]. This means that the key to the creation of this stacking fault structure is not the type of initial defect, but the initial defect provides the dislocation nucleation site.

**Figure 6:**
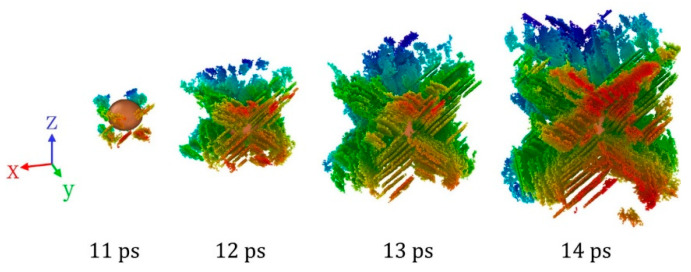
Dislocation and stacking fault structure near Cu inclusions at different times when *u_p_* = 1.5 km/s. The atoms are rendered by Y positions to enhance the stereoscopic effects.

When *u_p_* = 1.5 km/s, for the case of single crystal Al, the lattice is subjected to a large uniaxial strain along the [100] direction after the shock wave propagates, leading to the lattice structure at this time that is close to the ideal BCC structure [59], and the internal shear stress is too low to generate dislocation and stacking fault. However, when there are inclusions in the matrix, on the one hand, the interface of the inclusions provides the initial location for dislocation nucleation, and on the other hand, the interaction between the inclusions and the shock waves changes the stress state near the inclusions, making it easier for dislocation to be generated. Unlike the stacking fault structure that grows by dislocation slip at *u_p_* = 0.8 km/s, as shown in Figure 6, the expansion of stacking fault structure is accompanied by the continuous emergence of new stacking fault surfaces. This is also related to the high uniaxial strain in the single crystal after the shock wave propagates. When the stacking fault structure is generated at the inclusion interface, these new stacking fault structures will affect the surrounding stress–strain state, resulting in the continuous generation of new stacking fault surfaces, which will eventually extend to the whole shock compression region. For the case of *u_p_* = 2.0 km/s, the higher piston velocity makes the lattice transform into the ideal BCC structure with a more stable state, so the inclusions will not lead to the generation of a stacking fault structure.

### 3.3. Characteristics of Spall Damage at Low Piston Velocities

**Figure 7:**
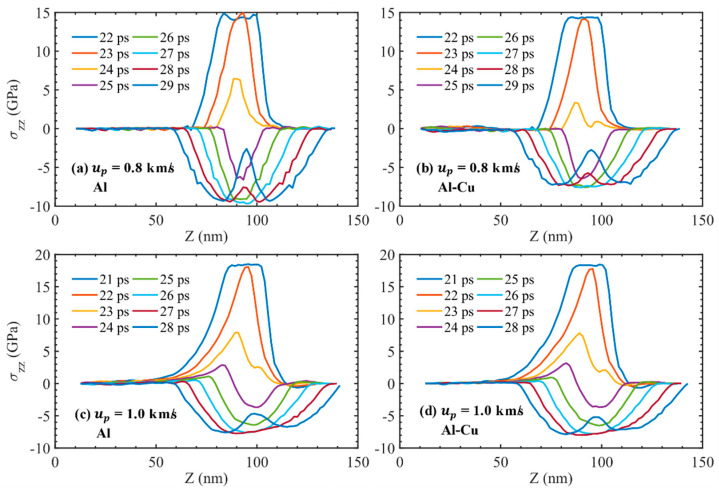
Evolution of longitudinal stress components for pure single crystal Al or the crystal with Cu inclusion at different piston velocities. (**a**) Al, *u_p_* = 0.8 km/s; (**b**) Al–Cu, *u_p_* = 0.8 km/s; (**c**) Al, *u_p_* = 1.0 km/s; (**d**) Al–Cu, *u_p_* = 1.0 km/s.

After the shock wave encounters the free surface, the interaction of its reflected sparse wave and the unloaded wave after the loading wave leads to the reduction of compressive stress and the formation of a tensile region. The evolution of longitudinal stress components for pure single crystal Al or the crystal with Cu inclusion are shown in Figure 7. Two piston velocities, *u_p_* = 0.8 km/s and *u_p_* = 1.0 km/s, were selected to represent the cases where the inclusions have an effect on the spall strength or have no effect, respectively. Comparing the stress evolution of ideal single crystal Al and single crystal Al with inclusions at *u_p_* = 0.8 km/s in Figure 7a,b, the main difference is found at 26 and 27 ps. In single crystal Al with inclusions, a plateau appears at the bottom of the stress distribution curve, which is due to plastic deformation in the tensile region causing the tensile stress to no longer increase. Nevertheless, for ideal single crystal Al, the tensile stress increases until fracture. This suggests that the inclusions can induce plastic deformation in the Al matrix, thus reducing the maximum tensile stress during tension. When *u_p_* = 1.0 km/s, both single crystal Al with inclusions and ideal single crystal Al have a large number of dislocations during the shock compression process, which also makes them prone to plastic deformation during the tensile process. As can be seen in Figure 7c,d, inclusions have almost no effect on the evolution of stress during the tensile fracture process.

**Figure 8:**
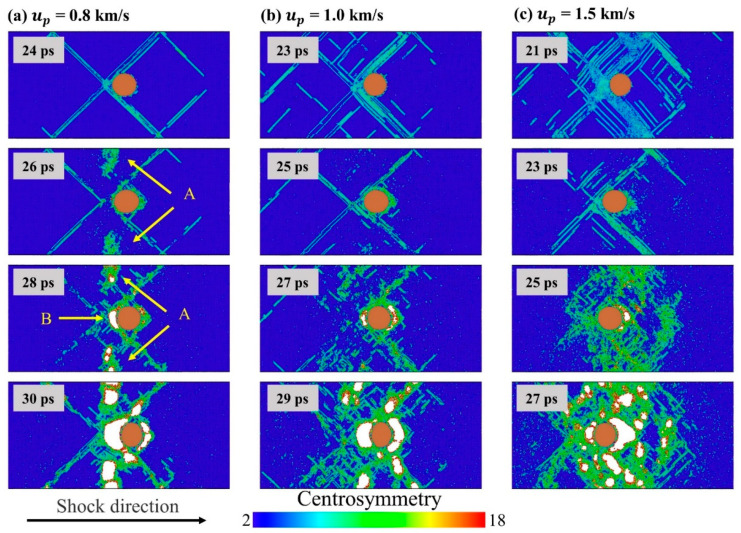
Microstructure evolutions of Al–Cu during spallation process. The Al atoms are color-coded by CSP. (**a**) *u_p_* = 0.8 km/s; (**b**) *u_p_* = 1.0 km/s; (**c**) *u_p_* = 1.5 km/s.

However, even if the inclusion itself has little effect on the stress evolution and spallation strength, it always has an important influence on the evolution of microstructure and the development of damage. Figure 8a–c shows the microstructure evolution of the tensile fracture process for single crystal Al with inclusions at *u_p_* = 0.8 km/s, 1.0 km/s, and 1.5 km/s, respectively. The Al atoms are color-coded by CSP analysis, which helps to determine local defects such as stacking faults (green regions) and void surfaces (red regions). Generally, for the case of ideal single crystal, the dislocation and stacking fault structure generated by the shock compression process will be greatly reduced during the unloading and tensile process, and few residual defects are retained [33,60]. However, for single crystal Al containing Cu inclusions, as shown in Figure 8, the stacking faults on the slip plane near the inclusions are more difficult to recover than dislocation and stacking faults in the matrix. This is due to the fact that the interface of the inclusions allows dislocations to exist stably. Besides, at all three different piston velocities, the voids preferentially nucleate near the interface of the inclusions, and grow along the interface of inclusions. This is consistent with the experimental understanding that voids will preferentially nucleate at the inclusions interface [14,21]. Even if the number of voids in the matrix increases with increasing piston velocity, the distribution of voids in the matrix will always have a clear symmetry.

**Figure 9:**
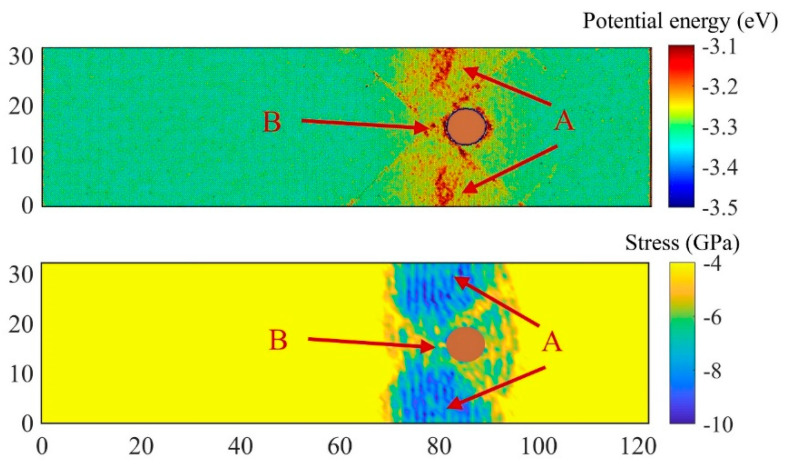
Spatial distributions of longitudinal stress components and atomic potential energy in the Al matrix in the central section of the system, *u_p_* = 0.8 km/s, t = 26 ps. Regions A and B here correspond to regions A and B in Figure 8a.

Take the case of *u_p_* = 0.8 km/s as an example for detailed analysis. As shown in Figure 8a, with the increase of tensile stress, a large number of defects appear in region A at t = 26 ps. Considering the periodic boundary, region A represents the middle region of the two inclusions. Then, at 28 ps, voids appear in region A and region B. Region B is the intersection of the four slip planes and is also the interface of inclusions. The void in region B is slightly larger than that in region A, indicating that the void nucleation at the inclusion interface is a little earlier than the matrix. During the subsequent growth process, the void at the inclusion interface is always the largest. In order to analyze the reason why voids preferentially nucleate in the A and B regions, we calculated the stress and potential energy distribution inside the matrix at the time before void nucleation, i.e., at t = 26 ps, as show in Figure 9. It can be seen that the stacking fault structure and Cu inclusions had significant effect on the distribution of potential energy and stress. The potential energies of regions A and B are high, which is consistent with the distribution of voids. However, due to the dislocation slip that will release stress, the stress in region B where the four slip planes intersect is lower, which is exactly opposite of the stress concentration near the inclusion during dynamic tensile process at a low strain rate [42]. In contrast, region A has a higher tensile stress, which also makes the atoms in region A have higher potential energy. In region B, the local atomic potential energy is high due to the interface of inclusions and dense defects. In summary, the preferential nucleation of voids in region A is due to the high potential energy caused by the high local tensile stress, while the preferential nucleation of voids in region B is due to the high potential energy of the inclusion interface and dense defects.

**Figure 10:**
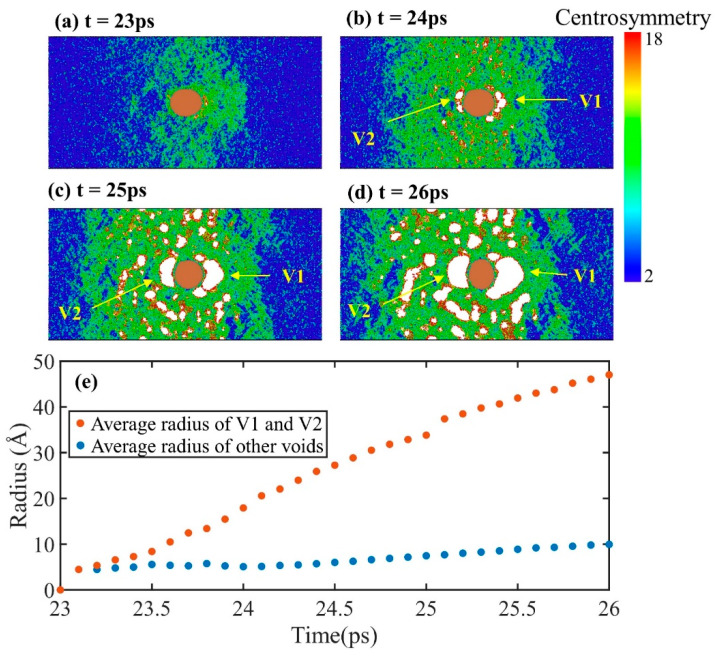
Microstructure evolutions of Al–Cu during spallation at *u_p_* = 2.0 km/s. The atoms are color-coded by CSP. (**a**) t = 23 ps; (**b**) t = 24 ps; (**c**) t = 25 ps; (**d**) t = 26 ps. (**e**) Evolution of the average radius of voids V1 and V2 and the average radius of other voids over time.

For the case of *u_p_* = 2.0 km/s, as shown in Figure 10, different from the cases of lower piston velocities, there is no dislocation in the tensile fracture process. On the one hand, this is due to the high strain rate induced by high piston velocity; on the other hand, there is no dislocation stacking fault in the matrix during impact compression. The absence of dislocations during tensile fracture process results in a random distribution of voids in the matrix., rather than a symmetrical distribution as at low piston velocities. When t = 24 ps, there are two obvious voids, V1 and V2, on the inclusion interface, and many small voids are randomly distributed in the matrix. The voids V1 and V2 grow along the boundary of the inclusion and grow faster than other voids. In fact, a similar phenomenon is observed at the lower piston velocities as shown in Figure 8. In this case, we calculated the equivalent radii of voids V1 and V2 and the averaged equivalent radii of other voids to quantify the growth of voids. The equivalent radius is calculated through the volume of the void:

(3) $$R_{eq}=\sqrt[{3}]{3V/4\pi }$$

where *V* is the volume of each void. The averaged radius at different times is shown in Figure 10e. The growth rate of the void radius at the inclusion interface is about five times larger than that of voids in the matrix. However, for pure Al without inclusions, the growth rate of the largest void is roughly the same as that of other voids in the early stage of damage [61]. This demonstrates that the interface between matrix and inclusion accelerates the growth of voids. This also explains why inclusions are observed at the bases of dimples in ductile transgranular fracture surfaces in spallation experiments [20,21,22].

### 3.4. Microstrucsture during Shock-Induced Spall Process at High Piston Velocities

**Figure 11:**
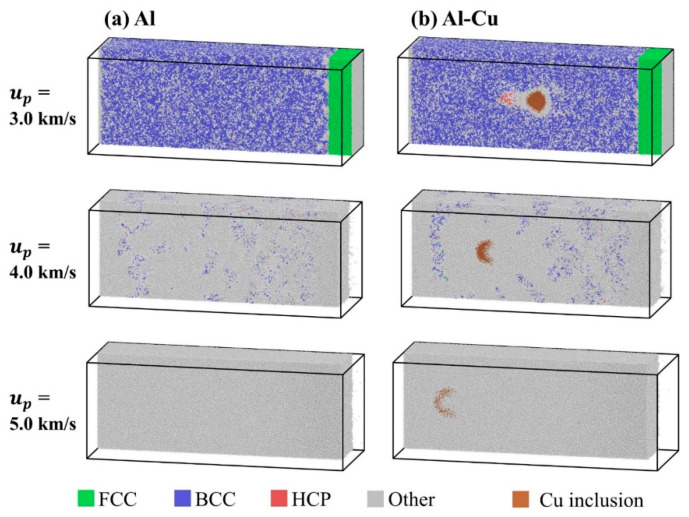
Atomic configurations during shock compression at 12 ps for different piston velocities as indicated on the left side. Al atoms are color coded by adaptive common neighbor analysis (a-CNA). (**a**) Ideal single crystal Al; (**b**) The crystal with Cu inclusion.

Figure 11 shows the shock compression microstructure of ideal single crystal Al and single crystal Al with inclusions at the *u_p_* = 3.0, 4.0, and 5.0 km/s. When *u_p_* = 3.0 km/s, the influence of inclusions on the microstructure of the matrix during shock compression is localized, and it only has a great influence near the interface of inclusion. The sample near the inclusion interface is completely transformed into amorphous structure, and a small number of atoms belonging to HCP structure appears at the back of the inclusion, which may be due to the reflection of the shock wave from the inclusion. When *u_p_* = 4.0 km/s, for both ideal single crystal Al and single crystal Al with inclusions, the state of the sample transforms into a mixture of complete melting region (all atoms are amorphous) and partial melting region (some of the atoms are amorphous) after shock wave propagates. In single crystal aluminum containing inclusions, the distribution of the partial melting region is symmetrical. In addition, at this piston velocity, the Cu inclusions are completely melted. As the Cu atoms are heavier compared to the Al atoms, it is more difficult for the thicker middle part of the spherical Cu inclusions to be pushed backwards. As a result, the Cu inclusion changes from the original spherical shape to a bowl-like shape. At the higher *u_p_* of 5.0 km/s, the Al matrix and Cu inclusions have completely melted, and they are almost completely mixed together. The deformation of inclusions is more significant. Experimentally, very similar particle shape changes were seen on the impact process of the polymer containing tungsten particle by in situ radiography [62].

**Figure 12:**
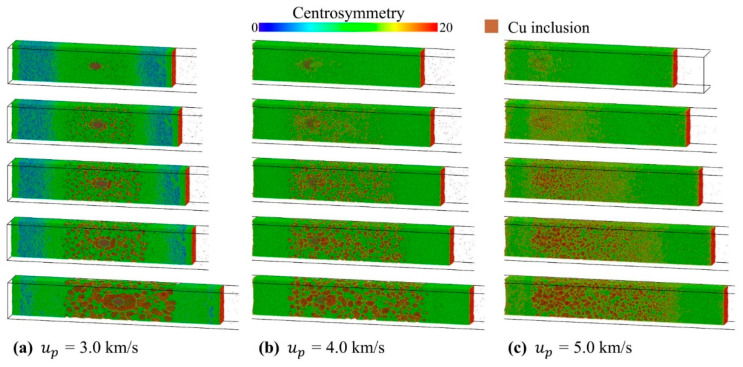
Microstructure evolutions of Al–Cu during spallation at different piston velocities. (**a**) *u_p_* = 3.0 km/s; (**b**) *u_p_* = 4.0 km/s; (**c**) *u_p_* = 5.0 km/s. The atoms are color-coded by CSP.

During the tensile fracture process, as shown in Figure 3, the inclusions have almost no effect on the spall strength for *u_p_* greater than 2.5 km/s. This is due to the fact that under high piston velocity (*u_p_* > 2.0 km/s), the material will undergo releasing melting or even compression melting, resulting in micro spallation [63]. As can be seen in Figure 12, a large number of voids nucleate simultaneously in the damage region. In addition, it is worth noting that for the case of *u_p_* = 3.0 km/s, the interface between inclusions and matrix is obvious. Although the interface of the inclusions is no longer the preferred location for voids nucleation, in the subsequent evolution, the voids tend to grow along the inclusions interface. For the case of *u_p_* = 4.0 km/s, due to the mutual diffusion between Al matrix and Cu inclusions, the feature that voids grow along the inclusions interface is not clear enough. When *u_p_* = 5.0 km/s, it is almost impossible to distinguish the influence of inclusions on void growth.

## 4. Conclusions

In this study, we used molecular dynamics to simulate the spall process of single crystal Al containing spherical Cu inclusions and investigated the effects of inclusions on the shock compression structure, spall strength, and damage evolution at different piston velocities. The free surface velocities and the statistics on the maximum tensile stress of the spall process showed that the presence of copper inclusions reduced the critical piston velocity required for spall to occur. The presence of inclusions significantly reduced the spall strength at 0.6 km/s ≤ *u_p_* ≤ 0.9 km/s and 1.5 km/s ≤ *u_p_* ≤ 2.0 km/s. This is due to the fact that in these two velocity ranges, the shock compression process of ideal single crystal Al did not produce dislocation and stacking fault. However, for the case of single crystal Al containing inclusions, due to the interference and reflection of inclusions on the shock wave and the initial nucleation position of dislocations provided by the inclusion interface, the dislocation and stacking fault structure appeared in the shock compression process. These defects promoted the plastic deformation of the matrix and the heterogeneous nucleation of voids in the tensile fracture process, and they finally reduced the spall strength. When 0.9 km/s ≤ *u_p_* ≤ 1.4 km/s, there were a lot of dislocations and stacking faults in both ideal single crystal Al and single crystal Al with Cu inclusions during shock compression process. When *u_p_* ≥ 2.5 km/s, the Al matrix would shock melt or unloading melt, and micro-spallation occurred. In these two velocity ranges, inclusions had no effect on spall strength.

Microstructural analysis of shock compression process revealed that when *u_p_* ≤ 0.9 km/s, inclusions led to the formation of a regular stacking fault structure composed of four stacking fault planes in the Al matrix. This stacking fault structure was grown by dislocation slip. When *u_p_* = 1.5 km/s, a more complex regular stacking fault structure appeared in single crystal Al containing inclusions; the expansion of this stacking fault structure was accompanied by the continuous emergence of new stacking fault planes. At a higher piston velocity (*u_p_* ≥ 2.0 km/s), the effect of inclusions on the microstructure of matrix during impact compression was reduced. In addition, for copper inclusions, when *u_p_* ≥ 4.0 km/s, Cu inclusions melted completely during shock compression process. Cu inclusion changed from the original spherical shape to a bowl-like shape.

Analysis of the microstructural evolution during the tensile fracture process showed that, as in the experiments and existing simulations, when classical spallation occurred (*u_p_* ≤ 2.0 km/s), voids preferentially nucleated at the interface of the inclusions due to the higher atomic potential energy. These voids grew along the interface at a rate five times faster than other voids. When *u_p_* ≥ 2.5 km/s, the Al matrix had undergone releasing melting or compression melting. The tensile fracture mode changed from classical spallation to micro spallation. Voids did not nucleate preferentially on the inclusion interface. Since Cu inclusions and Al matrix were not fully mixed, the voids also tended to grow and coalescence along the inclusion interface to form a large void. When *u_p_* = 5.0 km/s, the inclusions and matrix were fully mixed, and there was no significant effect on the nucleation or the evolution of voids. We believe that these simulation and analysis results can help establish the theoretical model and the design of the experiment. At the same time, we hope that there will be relevant experiments in the future to directly verify these simulation results.

## Figures and Tables

**Table 1:** Spall strengths given by the maximum tensile stress ($\sigma_{\mathrm{sp}}^{}$) and Equation (1) ($\sigma_{\mathrm{sp}}^{*}$). Strain rate $\dot{\varepsilon }$ given by Equation (2).

*u_p_* (km/s)	$\sigma_{sp}\;(\mathrm{GPa})$		$\sigma_{sp}^{*}\;(\mathrm{GPa})$		$\dot{\varepsilon }$ $\;(10^{10}\;s^{-1})$	
	Al	Al–Cu	Al	Al–Cu	Al	Al–Cu
0.6		7.58		8.36		1.81
0.7	9.13	7.79	10.91	7.98	1.35	1.78
0.8	9.71	7.64	10.26	7.81	1.87	1.72
0.9	7.68	7.56	8.16	7.73	1.8	1.68
1	7.8	7.76	7.93	7.44	1.49	1.55
1.1	7.89	7.81	7.77	7.8	1.48	1.31
1.2	7.88	7.71	7.84	7.56	1.49	1.3
1.3	8.09	7.8	7.79	7.39	1.49	1.31
1.4	7.97	7.84	7.95	7.15	1.61	1.49
1.5	9.58	7.71	9.5	7.45	1.73	1.35
2	8.11	7.76	8.06	7.44	1.51	1.51
2.5	6.74	6.67	6.13	6.18	1.00	1.01
3	5.72	5.67	5.89	5.92	1.01	1.04
4	4.59	4.57	5.55	5.31	1.01	1.06
5	3.5	3.47	4.48	4.09	0.83	0.78

## Data Availability

The data presented in this study are available on request from the corresponding author.
